# The impact of economic complexity on embodied carbon emission in trade: new empirical evidence from cross-country panel data

**DOI:** 10.1007/s11356-021-14414-3

**Published:** 2021-05-27

**Authors:** Muhammad Qayyum, Yuyuan Yu, Shijie Li

**Affiliations:** 1 School of Economics, Hainan University, Haikou City, Hainan Province China; 2 School of International Economics and Trade, Central University of Finance and Economics, Changping District, Beijing, China

**Keywords:** Economic complexity, Embodied carbon emission in trade, GMM, Trade and environment, Panel data, C33, Q56, O11, P51

## Abstract

Establishing a fair platform for allocating carbon emission responsibility worldwide determines the sustainability and efficiency of the world’s climate policy and framework. In the context of global environmental load displacement and CO_2_ transfer, this paper endeavors to examine the relationship between economic complexity and embodied carbon emissions based on cross-country panel data. Our study utilizes the generalized method of moments (GMM) approach to estimate our dynamic models covering 34 OECD countries and 24 non-OECD countries from 1995 to 2015. The empirical results show a heterogeneous impact of economic complexity on embodied carbon emissions in exports (EEE) and imports (EEI). Besides, the scale effect, composition effect, and technology effect are also significant drivers of embodied carbon emissions. The improvement of economic complexity can decrease the marginal effects of export scale and export structure on foreign EEE (but not domestic EEE) significantly, while the marginal positive impacts of technology on EEE can be further enhanced by economic complexity growth. Moreover, there is no strong evidence to prove the significant indirect impacts of economic complexity on foreign carbon emission embodied in imports, while economic complexity has significantly positive indirect impacts on domestic carbon emission embodied in imports only through import scale. In the subsample regressions, we found asymmetric impacts of economic complexity between high-income countries and low- and middle-income countries.

## Introduction

Economic globalization has greatly facilitated the development of the world economy by allocating productive resources worldwide efficiently, such as labor, capital, and technology. However, the extension of the global value chain (GVC) has also resulted in the so-called environmental load displacement or CO_2_ leakage, which is defined as the transboundary flow of pollutants and “displaced” environmental degradation from the consumption-based view (Muradian et al. 2002). Under the GVC system, some developing countries at the low end of the GVC division tend to undertake the pollution-intensive industries from developed countries and suffer from serious environmental problems. The principle of producer responsibility in the present global climate policy framework fails to account for the imbalance between foreign consumption and local environmental degradation in some developing countries (Muradian et al. 2002; Fei et al. 2020). Therefore, it is crucial to calculate the carbon emission embodied in trade (EET), which is defined as the sum of direct and indirect carbon emissions in goods’ production and trade process (Peters and Hertwich 2008). To achieve worldwide sustainable development is also one of the crucial aspects of green development, which refers to a modern mode of development established under the challenges of natural energy and resource management capacity, and recognizes sustainable development through environmental protection (Fang et al. 2020c). An efficient carbon trading platform is crucial to the cost reduction of pollution prevention and the realization of integration between environmental and economic benefits (Zhong et al. 2018; Fang et al. 2020a).

With the deepening and broadening of GVC, the volume of carbon emissions embodied in exports (EEE) or imports (EEI) is significant in OECD countries and four developing countries (Brazil, China, India, and Russia). Among these countries, EEE or EEI is usually 10–20% higher than domestic production (Ahmad and Wyckoff 2003). Besides, it has been estimated that the size of the global CO_2_ leakage increased by nearly 80% from 1995 to 2007 (Sato 2014; Lenzen 2016). Assessing the EET and its determinants has attracted much attention in the academic field in recent years. By using various decomposition techniques based on the input-output tables, many studies have calculated the EET for individual countries, such as Machado et al. (2001) for Brazil, Sánchez-Chóliz and Duarte (2004) for Spain, Peters and Hertwich (2006) for Norway, Mäenpää and Siikavirta (2007) for Finland, Su et al. (2017) for Singapore, Wang et al. (2018) for India, and Du et al. (2020) for China. Meanwhile, some studies have attempted to examine the driving factors of EET. For example, EET could vary with GDP (Islam et al. 2016), total population (Islam et al. 2016; Huang and Zhao 2018), technological level (Wang et al. 2020), trade openness (Islam et al. 2016), trade structure (Wang et al. 2019), financial development (Huang and Zhao 2018), trade in intermediate goods (Fei et al. 2020), and so on. However, as far as we know, no study has examined the determinants of EET comprehensively at the empirical level. Besides, a multidimensional evaluation system should be established to capture the complexity of the environmental system (Fang et al. 2020b). In this context, researchers should provide more insights on how to control EET and allocate the responsibility for carbon emissions worldwide.

As an indicator evaluating economic development, economic complexity is often considered by policymakers when they shape national economic and energy policies (Neagu and Teodoru 2019; Yilanci and Pata 2020; Yu and Qayyum 2021). Developed by Hidalgo and Hausmann (2009), the economic complexity index (ECI) captures each country’s capability in producing goods. Countries with higher ECI can produce more complex and knowledge-based products, thus indicating a more advantageous product space (Yu and Qayyum 2021). On the contrary, in simpler economies, individuals and firms produce a fewer range of products with less knowledge. Therefore, in some studies examining the environmental impacts of ECI, ECI is often connected to economic development (Yilanci and Pata 2020) or structural transformation (Doğan et al. 2020). However, existing studies found conflicting results about the nexus between economic complexity and carbon emissions. A suppressing effect of ECI growth has been found on carbon emissions by Can and Gozgor (2017) and Doğan et al. (2020). Based on different sample and estimation techniques, more researchers found that the increase in ECI may further deteriorate environmental quality (Lapatinas et al. 2019; Neagu and Teodoru 2019; Yilanci and Pata 2020). Some researchers even found an inverted U-shaped relationship between economic complexity and carbon emissions (Dogan et al. 2019; Neagu 2019).

Even though the relationship between economic complexity and carbon emission has been analyzed extensively, there is a huge research gap on linking economic complexity (or economic transformation) with carbon embodiment in trade. Therefore, one of the key research problems of this paper is to examine the direct impacts of economic complexity on a set of decomposed EET indicators. Besides, as Grossman and Krueger (1991) dissected the determinants of EET into scale effect, composition effect (or the structural effect), and technology effect (or the emission intensity effect), another key research problem of this paper is to analyze how economic complexity affects EET indirectly through these channels. Intuitively, the increase in ECI often indicates the structural transition towards a more industrialized and knowledge-based economy. The industrialization of an economy also accompanies accelerating energy consumption, thus increasing local carbon emissions (Bai et al. 2020). Therefore, countries with higher ECI could experience excessive environmental degradation due to the expansion of more diverse and complex production and goods (Swart and Brinkmann 2020). By contrast, countries with lower ECI mainly focus on the procession of intermediate goods and raw material or agricultural goods production. Therefore, the decline in environmental quality in simpler economies is limited. Countries being “pollution heaven” can alleviate environmental degradation by developing more environmental-friendly technologies and producing cleaner products (Yilanci and Pata 2020).

From the above argument, it is necessary to examine the nexus between economic complexity and EET based on cross-country panel data. The nexus between economic complexity and EET has been indefinite. Therefore, this paper contributes to the existing literature in the following two aspects: (i) To the best of our knowledge, this paper takes the first attempt to examine the relationship between economic complexity in EET based on cross-country panel data. (ii) This paper, for the first time, utilizes a set of decomposed EET indicators as dependent variables and tests their determinants by using the generalized method of moments (GMM) technique. For these two reasons, we expect that the empirical findings of this paper would enrich the existing literature in environmental economics.

The remainder of this paper proceeds as follows: the “Literature review and theoretical analysis” section summarizes and compares the existing literature and establishes theoretical foundation; the “Data and methodology” section introduces the data source and methodological framework; the “Findings and discussionS6” section contains the estimation results and analysis; and the “Conclusions” section concludes the whole paper and provides some policy implications.

## Literature review and theoretical analysis

### Literature review

#### Nexus between economic complexity and carbon emissions

In recent years, an increasing number of studies examine the environmental impacts of economic complexity. There is no definite conclusion about the relationship between economic complexity and carbon emissions. Researchers draw opposite conclusions based on different sample data and estimation techniques. Existing research found three possible effects of economic complexity on carbon emission: positive impacts (the increased economic complexity could lead to environmental degradation), negative impacts (the increased economic complexity could improve environmental quality), and an inverted U-shaped relationship.

The first empirical study that tests the nexus between economic complexity and carbon emission was conducted by Can and Gozgor (2017) based on France’s time-series data from 1964 to 2014. Their DOLS (dynamic ordinary least squares) estimation results show that economic complexity has a suppressing capacity on France’s carbon emission. Based on the panel data for 28 OECD countries in 1990–2014, Doğan et al. (2020) used various estimation techniques like augmented mean group (AMG), FMOLS (full modified ordinary least square), DOLS, panel ARDL (autoregressive distributed lagged model), and fixed effect method to test the liaison between economic complexity and carbon emission. They also concluded that an increase in renewable energy consumption and ECI, as structural economic transformation towards more knowledge-based production, could mitigate further environmental degradation problems for developed countries like OECD members. After establishing a Product Emission Intensity Index (PEII) for 67 countries between 1976 and 2012, Romero and Gramkow (2021) concluded that a 0.1-unit increase in the economic complexity index could lead to a 2% decrease in carbon emission of the next period.

However, other scholars reached opposite conclusions based on the different sample and estimation techniques. In a country-panel setting covering 88 countries from 2002 to 2012, Lapatinas et al. (2019) adopted the fixed-effects 2SLS (two-stage least squares) method and concluded that the negative impact of the economic complexity on air quality is robust and significant. In other words, it is more likely that countries that produce more complex goods are exposed to inferior air quality (higher carbon emission or PM2.5). This conclusion is also applicable to the case of 25 EU (European Union) members for the period of 1995–2016. Based on FMOLS and DOLS techniques, the empirical research of Neagu and Teodoru (2019) shows that countries with higher ECI are faced with higher risks of pollution. Using the time-series data of China from 1965 to 2016, Yilanci and Pata (2020) utilized the Fourier ARDL procedure to test the validity of the EKC hypothesis in China, that is, the relationship between economic development and environmental performance. Their empirical results indicate that the EKC hypothesis does not hold in China’s case, and economic complexity could impede environmental quality in the short term and long term because China’s current economic complexity does not encourage green technology.

Some scholars found an inverted U-shaped relationship between economic complexity and carbon emissions. Neagu (2019) confirmed this conclusion by employing the cointegrating polynomial regression, panel FMOLS, and DOLS techniques based on panel data of 25 selected EU countries in 1995–2017. He pointed out that the increase in carbon emissions in the first stage of economic complexity advances could be resulted from the dominance of the scale effect, that is, a larger proportion of resource and energy consumption embedded in the production of more complex and sophisticated goods. However, in the second stage, a higher economic complexity could suppress carbon emissions due to the dominance of technological effect, that is, higher efficiency in energy use. Dogan et al. (2019) also reached the same conclusion by applying a panel quantile regression approach to selected panel data covering 55 countries in 1971–2014. They pointed out that economic complexity enhanced carbon emissions in lower-middle and higher middle-income countries while controlling carbon emissions in high-income countries. Pata (2021) also concluded that an inverted U-shaped EKC relationship is valid in the case of the USA between economic complexity and environmental pollution based on the combined cointegration test and three different estimators for the period from 1980 to 2016.

#### Research on the determinants of EET

Grossman and Krueger (1991) dissected the determinants of air pollution into scale effect, composition effect (or the structural effect), and technology effect (or the emission intensity effect). To be specific, the scale effect captures the simple intuition that the expansion of economic activity could increase the total amount of pollution. The structural effect regulates that changes in production or consumption structure have ambiguous impacts on environmental quality because it is not sure whether the country’s production or consumption would become more pollution-intensive. Lastly, the technology effect states that technological advancement could reduce pollution per unit of output or pollution intensity. Based on this framework, in recent years, many researchers have studied the determinants of carbon emissions embodied in trade by more advanced decomposition methods based on three popular environmental input-output analysis (IOA), which are single region input-output (SRIO), bilateral trade input-output (BTIO), and multi-region input-output (MRIO) models (Sato 2014). Xu and Dietzenbacher (2014) applied structural decomposition analysis (SDA) to estimate the embodied carbon emission in 40 countries from 1995 to 2007, and the estimation results confirmed the importance of structural effect and emission intensity effect to the changes in EET. By performing index decomposition analysis (IDA), Dong et al. (2010) and Wu et al. (2016) used the data of carbon emission and China-Japanese trade between 1990–2000 and 2000–2009, respectively. They concluded that the decrease in emission intensity was a strong driver for the decrease of embodied caron emission, while growth in trade volume acted as a major driving force for the growth of embodied emission. Su et al. (2017) conducted the first comprehensive analysis of Singapore’s embodied carbon emissions by using SDA based on IOA. Their findings show that the scale effect (the expansion of export volume and export-oriented industries) and emission intensity effect (energy efficiency) are significant driving factors for the changes in Singapore’s emissions. Based on the MRIO framework and spatial econometric models, Zhong et al. (2018) unraveled the spatial carbon emission interchanges for 39 economies from 1995 to 2011. They concluded that the energy and industrial structure have spatial spillover effects on EET, while the impact of energy efficiency is not significant because the market responses could offset the decrease in carbon emission resulted from improved energy efficiency. Wang et al. (2019) decomposed the embodied carbon emission in China-German trade from 1995 to 2009 at the sectoral level by using SDA. They pointed out that the emission intensity effect acted as the most prominent driving force for reducing the net EET, followed by the structural effect of German’s final demand and intermediate input and the scale effect of China’s final demand.

Some researchers have added other variables into their econometric models and reached interesting conclusions. Islam et al. (2016) tested the impact of trade openness on embodied carbon emission by employing the fixed-effect model based on sizeable country-level panel data covering 187 countries in 1990–2011. Their empirical results show that a one-unit increase in trade openness could lead to a 10–23% increase in EET, while GDP has positive impacts on EEE (to increase embodied emissions in exports) and negative impacts on EEI (to decrease embodied emissions in imports). After measuring the EEE for China based on the MRIO model for the period of 1995–2011, Fei et al. (2020) found that trade in intermediate goods was a driving factor for the fast growth of emission embodied in China’s exports. Besides, with the deepening of GVC (global value chain) participation, the positive impact of the GVC division on EEE became even stronger. Based on STIRPAT (Stochastic Impacts by Regression on Population, Affluence and Technology) model, the empirical results of Wang et al. (2020) confirmed the importance of technological progress to embodied carbon emission in China’s high- and new-technology industries.

### Theoretical analysis

**Fig. 1:**
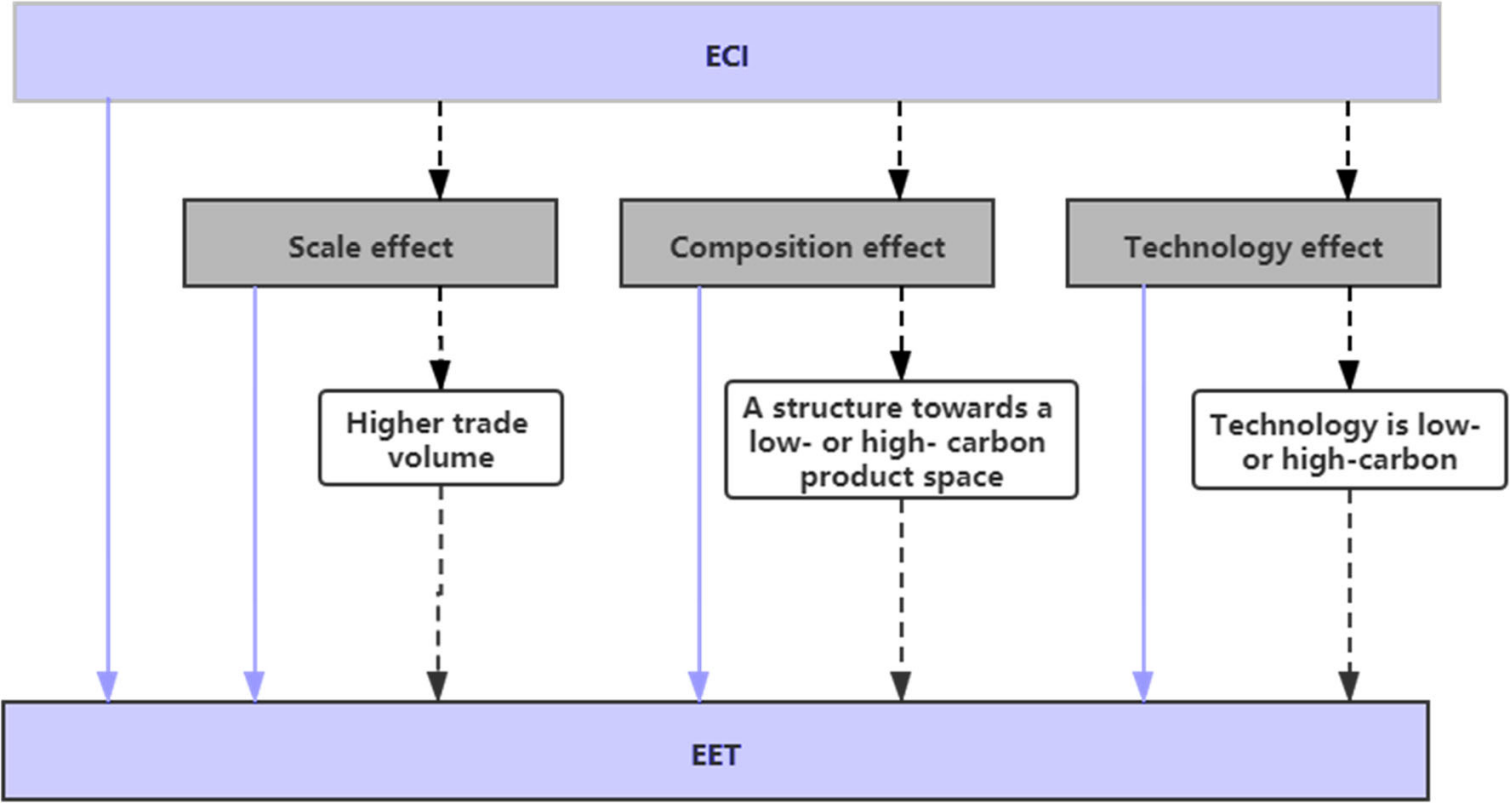
Impact mechanism of economic complexity on EET

Based on the above analysis of existing literature, it can be concluded that the scale effect, composition effect, and technology effect are primary influencing factors of EET. As an important indicator of economic transformation, economic complexity may exert its impact on EET directly or indirectly through the above three effects (see Fig. 1). Higher economic complexity or a more knowledge-based production system may have ambiguous impacts on EET together with scale effect, composition effect, technology effect, and other factors. Besides, economic complexity also has growth effects as countries with higher economic complexity have better GDP performance (Hartmann et al. 2017), which could lead to larger trade volume, thus affecting EET indirectly. The second indirect influence channel of economic complexity is through the composition effect. Dynamic changes in product space often cause structural changes in the products that one country exports or imports. The final influencing direction is dependent on the overall carbon intensity after structural changes in product space. Another distinct influencing mechanism consists in the technology effect. A more complex production system is often the result of production capability improvement and technology progress. Similarly, the final impact of this channel relies on whether the improved technology contains low carbon intensity or high carbon intensity. Therefore, this study proposes the following hypotheses for empirical tests:

- **Hypothesis a:** Economic complexity has significant direct impacts on EET after controlling the scale effect, composition effect, technology effect, and other factors.
- **Hypothesis b:** Economic complexity will affect EET significantly and indirectly through the scale effect, composition effect, and technology effect.

## Data and methodology

### Data characteristics

This study aims to test the impact of economic complexity on trade-embodied carbon emission (million Tonnes) for 34 OECD countries and 24 non-OECD countries from 1995 to 2015. The selected countries account for nearly 90% of the world’s total GDP, 90% of the world’s total merchandise trade and 70% of the world’s total population in 2015. Therefore, sample countries covered in this paper can represent the basic economic patterns of most countries around the world. Sample countries included in this study are summarized in Table 8 in the Appendix.

Table 1 lists the variables employed in the paper and their detailed explanation. Specifically, our dependent variables, EEI and EEE, include a set of decomposed variables that measure the embodied carbon emission in export and import. A more detailed explanation of the dependent variables is shown in Table 9 in the Appendix. OECD Trade in Embodied CO_2_ Database provides extensive data on trade-embodied carbon emissions for various industries. Economic complexity is the core independent variable in this paper and the annual country-level data is available at OEC (The Observatory of Economic Complexity). The remaining variables in Table 1 are all explanatory variables that have been considered as the determinants of embodied carbon emissions by existing literature (Islam et al. 2016; Fei et al. 2020). These variables’ data source is from the World Bank database, WTO database, and WITS (World Integrated Trade Solution) database.

**Table 1:** Explanation of key variables

Variable	Explanation
EEI	Embodied emission in imports (in million tonnes)
EEE	Embodied emission in exports (in million tonnes)
eci	Economic complexity index
lnexport	Logarithm of annual merchandise exports (in million USD)
Ilnimports	Logarithm of annual merchandise imports(in million USD)
lnExportShare	Logarithm of manufactures exports (of total merchandise exports, %)
lnImportsShare	Logarithm of manufactures imports (of total merchandise imports, %)
lnExportsInter	Logarithm of intermediate goods exports (in thousand USD)
lnExportsInterShare	Logarithm of the share of intermediate goods exports (of total merchandise exports, %)
lnImportsInterShare	Logarithm of the share of intermediate goods imports (of total merchandise imports, %)
lnImportsInter	Logarithm of intermediate goods imports (in thousand USD)
lnAHSWeighted	Logarithm of the effectively applied weighted average tariff for intermediate goods (%)
lnPOP	Logarithm of the total population
lnGDPP	Logarithm of GDP per capita (in the current USA)
lnintensity	Logarithm of CO_2_ emissions intensity (kg per 2010 USD of GDP)
RD	Research and development expenditure (of GDP, %)

**Fig. 2:**
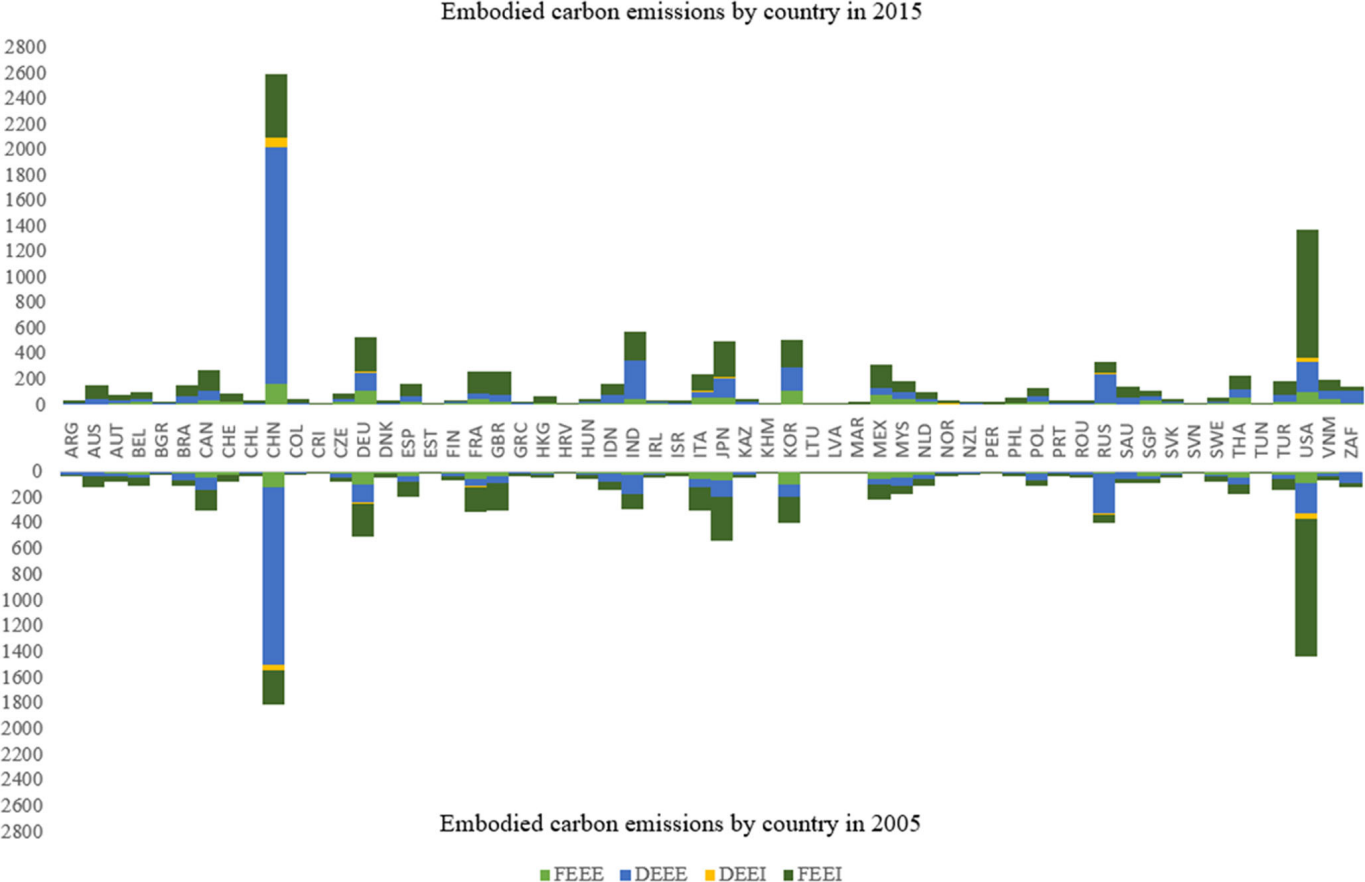
Composition of embodied carbon emission by country in 2015 (upper) and 2005 (down)

Figure 2 depicts the changes in the decomposed embodiment of carbon emissions from 2005 to 2015, including FEEE (foreign carbon emission embodied in exports), DEEE (domestic carbon emission embodied in exports), FEEI (foreign carbon emission embodied in imports), and DEEI (domestic carbon emission embodied in imports). More specifically, FEEE captures the CO_2_ emissions embodied in imported intermediate goods and services into a domestic industry. DEEE measures the CO_2_ emissions embodied in exports that have been generated anywhere in the domestic economy. FEEI captures the foreign CO_2_ emissions embodied in gross imports of country/region *c* with origin in exporting industry *i* in exporting country/region partner *p*. Similarly, DEEI captures the domestic CO_2_ emissions embodied in gross imports of country/region *c* with origin in exporting industry *i* in exporting country/region partner *p* (Wiebe and Yamano 2016). The sum of these variables evaluates the total trade-embodied carbon emission. In general, it can be seen that the top countries that contain high trade-embodied carbon emissions are China, India, the USA, Japan, South Korea, and Germany. Moreover, the total trade-embodied carbon emissions have increased a lot from 2005 to 2015 for developing countries like China and India. Besides, it can also be found that foreign carbon emissions embodied in imports account for a substantial part of its total EET for developed countries represented by the USA. However, developing countries represented by China have been exporting products that contain high domestic carbon emissions.

Table 2 summarizes the descriptive statistics of our explanatory variables. It illustrates that the variable of interest, ECI, which ranges from −1.5 to 2.5 with a mean value of 0.715 in the pooled sample, shows a much higher mean value for high-income countries than low- and middle-income countries. This indicates that high-income countries have more capacity to produce more complex and knowledge-based products than low- and middle-income countries on average in our sample. Besides, according to the mean value, high-income countries also show more advantage in other economic elements, such as import and export scale of both manufactured goods and intermediate goods, GDP per capita, and R&D. By contrast, low- and middle-income countries exhibit higher mean value in lower effective tariff for intermediate goods and total population.

**Table 2:** Descriptive statistics of explanatory variables

Variable	Pooled sample					Subsample (mean)	
	Mean	SD	Min	Max	N	High income	Low and middle incomes
eci	0.715	0.778	−1.476	2.468	638	1.084	.111
lnexport	11.60	1.348	8.037	14.67	638	11.844	11.193
lnExportShare	4.014	0.585	2.034	4.582	638	4.081	3.906
lnimports	11.65	1.262	8.276	14.70	638	11.874	11.276
lnImportsShare	4.234	0.137	3.746	4.519	638	4.232	4.237
lnExportsInter	16.91	1.461	11.58	19.75	638	17.193	16.457
lnExportsInterShare	3.042	0.505	0.765	3.967	638	3.155	2.856
lnImportsInter	17.10	1.212	14.36	19.91	638	17.214	16.907
lnImportsInterShare	3.091	0.247	2.163	4.034	638	3.008	3.226
lnAHSWeighted	1.352	0.435	−0.083	2.783	638	1.405	1.267
lnPOP	16.93	1.491	14.09	21.04	638	16.373	17.828
lnGDPP	9.623	1.127	6.161	11.54	638	10.332	8.464
lnintensity	−1.058	0.709	−2.878	0.535	638	−1.377	−.536
RD	1.350	1.064	−1.206	4.514	638	1.83	.564

### Regression framework

By referring to existing studies related to the driving factors of trade-embodied carbon emissions (Islam et al., 2016; Fei et al., 2020; Wang et al., 2020), a dynamic panel regression model that incorporates the lagged dependent variable has been established to examine the impact of economic complexity on EEE and EEI as Eqs. (1) and (2):

1 $$ \ln {EEE}_{it}={\alpha}_0+{\alpha}_1L.{EEE}_{it}+{\alpha}_2{ECI}_{it}+{\alpha}_3\ln {export}_{it}+{\alpha}_4\ln {ExportShare}_{it}+{\alpha}_5\ln {ExportInter}_{it}+{\alpha}_6\ln {ExportInter Share}_{it}+{\alpha}_7\ln intensity+{\alpha}_8\ln AHSWeighted+{\alpha}_9\ln POP+{\alpha}_{10}\ln GDPP+{\alpha}_{11}\ln RD+{\mu}_{it} $$

2 $$ \ln {EEI}_{it}={\beta}_0+{\beta}_1L.{EEE}_{it}+{\beta}_2{ECI}_{it}+{\beta}_3\ln {import}_{it}+{\beta}_4\ln {ImportShare}_{it}+{\beta}_5\ln {ImportInter}_{it}+{\beta}_6\ln {ImportInter Share}_{it}+{\beta}_7\ln intensity+{\beta}_8\ln AHSWeighted+{\beta}_9\ln POP+{\beta}_{10}\ln GDPP+{\beta}_{11}\ln RD+{\varepsilon}_{it} $$

In Eq. (1), the dependent variable, ln*EEE*_*it*_, represents the logarithm of embodied carbon emissions embodied in exports from country *i*to rest of the world in year *t*. This variable can be further decomposed into domestic carbon emission and foreign carbon emission embodied in exports, which are expressed as ln*FEEE*_*it*_ and ln*DEEE*_*it*_ in later regressions. Similarly, in Eq. (2), the dependent variable is embodied carbon emission in imports in the manufacturing industry. ln*EEI*_*it*_ measures the carbon emissions embodied in country *i*’s imports to the rest of the world in year *t*. OECD database also provides the decomposed data on this variable, including ln*FEEI*_*it*_ and ln*DEEI*_*it*_. *L*. *EEE*_*it*_ is the lagged one-period dependent variable, which is employed to observe the path dependence of embodied carbon emissions in the model. *ECI*_*it*_ is the variable of interest, and its estimated coefficient *α*_2_ and *β*_2_ measure the impact of economic complexity on EEE and EEI. ln*export*_*it*_, ln*import*_*it*_, ln*ExportInter*_*it*_, and ln*ImportInter*_*it*_ are the proxies for the scale of manufactured goods trade and intermediate goods trade. The estimated coefficients of ln*ExportShare*_*it*_, ln*ExportInterShare*_*it*_, ln*ImportShare*_*it*_, and ln*ImportInterShare*_*it*_ evaluate the structural effect of manufactured goods trade and intermediate goods trade, respectively. The remaining variables are all control variables, including ln*intensity*, ln*AHSWeighted*, ln*POP*, ln*GDPP*, and ln*RD* (see Table 2 for more details). Specifically, ln*intensity* can reflect the technological level while ln*AHSWeighted* measures local trade policy, the importance of which has been discussed by Wang et al. (2020) and Böhringer et al. (2017). Besides, ln*POP*, ln*GDPP*, and ln*RD* are typical variables included in STIRPAT models.

To test the possible impact mechanism, this paper has added relevant interaction terms between economic complexity, export/import, share of export/import, carbon intensity into Eqs. (1) and (2), including *eci* ∗ ln *export*, *eci* ∗ ln *import*, *eci* ∗ ln *ExportShare*, *eci* ∗ ln *ImportShare*, and *eci* ∗ ln *intensity*.

This paper employs the generalized method of moments (GMM) approach to estimate this dynamic model, which has been frequently used in empirical studies to avoid statistical problems like endogeneity, measurement error, and heteroscedasticity (Ganda 2019). Ganda (2019) also pointed out that the GMM approach is very suitable under the panel data setting that contains larger cross-sectional units (*N*) than the time period (*T*). This paper has the panel data of 58 countries (*N* = 58) from 2005 to 2015 (*T* = 11). Therefore, the GMM technique is appropriate for the estimation of Eqs. (1) and (2) when compared to other estimation techniques. The GMM method was initially developed by Arellano and Bond (1991) who resolved their econometric model by taking the first difference of the equation, also known as difference GMM. Later, based on this approach, Blundell and Bond (1998) obtained their system GMM estimators by using instrumental variables. However, the difference GMM approach assumed uncorrelated error terms and the variables are weakly endogenous. Therefore, this study will utilize the one-step system GMM approach because the one-step system GMM is believed to be more efficient, as suggested by Blundell and Bond (1998) and Bond (2002). We will use the Sargan tests to check the validity of instrumental variables and AR(1) and AR(2) to test residuals’ autocorrelation. GMM estimation assumes that the residuals have first-order autocorrelation but not second-order autocorrelation.

## Findings and discussion

### Full sample results

Table 3 lists the empirical results of Eqs. (1) and (2) based on the pooled sample. For EEE, the coefficients *ECI* are all significantly positive at the level of 5% in columns (1) and (2), indicating that a one-unit increase in economic complexity can result in a significant rise in FEEE and DEEE by 18.8% and 16.5%, respectively. Greater industrialization and economic development, represented by increasing economic complexity, have resulted in excessive environmental degradation in EEE, both domestic and foreign. In column (1), the coefficient of ln*export* and ln*ExportShare* is all positive and significant, indicating positive scale effect and structural effect of manufactured product exports on FEEE. Specifically, a 1% growth in the export of manufactured goods could increase FEEE by 0.356% significantly at the level of 5%, while a 1% rise in the share of manufactured goods could increase FEEE by 0.434% significantly at the level of 1%. As for intermediate goods, the estimated coefficient of ln*ExportInter* is positive and significant at a 1% significance level. This implies that the expansion of intermediate goods exports has a significant promoting effect on FEEE, which is also consistent with the prediction of Grossman and Krueger (1991). However, the structural effect of exporting intermediate goods is significant at the 1% level but is negative, implying that FEEE could be reduced significantly by 0.317% when the proportion of intermediate goods export increases by 1%. Ricci (2007) pointed out that intermediate goods related to more massive embodied emissions tend to contain higher productivity. Therefore, an increased export share of intermediate goods that are pollution-intensive could be a sign of the upstream movement of carbon leakage, thus reducing FEEE. In column (2), it can be seen that the DEEE is only significantly affected by the export scale of intermediate goods, but not the export scale, share of manufactured goods, and share of intermediate goods.

**Table 3:** Pooled sample regression results

	(1)	(2)	(3)	(4)
	lnFEEE	lnDEEE	lnFEEI	lnDEEI
eci	0.188**	0.165**	0.164***	0.035
	(2.42)	(2.49)	(2.92)	(1.53)
lnexport	0.356**	0.106		
	(2.47)	(1.51)		
lnExportShare	0.434***	−0.076		
	(4.31)	(−1.36)		
lnExportsInter	0.422***	0.255***		
	(2.58)	(3.75)		
lnExportsInterShare	−0.317*	−0.073		
	(−1.94)	(−1.25)		
lnimports			0.233**	0.004
			(2.20)	(0.08)
lnImportShare			0.430**	−0.017
			(2.54)	(−0.35)
lnImportsInter			0.038	0.018
			(0.38)	(0.42)
lnImportsInterShare			0.000	−0.055
			(0.00)	(−0.73)
lnintensity	0.150**	0.548***	0.153***	0.061**
	(2.16)	(6.83)	(3.09)	(2.01)
lnAHSWeighted	0.154***	0.133***	0.019	0.008
	(3.02)	(3.38)	(0.38)	(0.54)
lnPOP	−0.069	0.221***	0.364***	0.050*
	(−0.91)	(3.56)	(5.76)	(1.70)
lnGDPP	−0.195*	0.119	0.242***	0.016
	(−1.80)	(1.56)	(3.53)	(0.54)
RD	−0.004	0.007	−0.019	0.018
	(−0.09)	(0.26)	(−0.74)	(1.58)
Sargan test	272.81***	129.12***	310.42***	203.11***
AR(1)	0.002	0.000	0.000	0.001
AR(2)	0.064	0.648	0.061	0.034
_cons	−7.269***	−8.048***	−11.02***	−1.011
	(−4.22)	(−5.53)	(−6.60)	(−1.47)
*N*	580	580	580	580

1) ***, **, and * indicate that the coefficients are significant at the 1%, 5%, and 10% levels of significance, respectively. Numbers in brackets are *t* values

2) Sargan test checks the overidentification of instruments. Chi statistics are reported for the Sargan test, while the p-value is reported for AR(1) and AR(2)

In Table 3, it can be seen that the GMM estimator of *ECI* is positive and significant at the level of 1% in column (3), but not significant in column (4). This shows that economic complexity has a positive and significant impact on FEEI, while its impact on DEEI is statistically insignificant. With a larger comparative advantage in knowledge-based goods, the country can import more pollution-intensive products that are embodied with considerable foreign carbon emissions by exporting more complex goods. Besides, as can be seen from column (3), the coefficients of ln*import* and ln*ImportShare* are all positive and significant at the level of 5%, indicating positive scale effect and structural effect of manufactured goods on FEEI. Expressly, a 1% growth in the import scale of manufactured goods could increase FEEI by 0.233% significantly. Other aspects being equal, a 1% rise in the share of manufactured goods imports could increase FEEI by 0.43%. Besides, the estimated coefficients of ln*ImportInter* and ln*ImportInterShare* are positive but insignificant in statistics. Similarly, there is no sufficient evidence to conclude that DEEI is significantly affected by the scale effect or structural effect of manufactured goods imports and intermediate goods.

Furthermore, the technological effect, measured by carbon emission intensity, is significantly positive in columns (1)–(4). This means that a lower technological level, represented by higher carbon emission intensity per GDP, could further deteriorate the local environment by releasing higher embodied carbon emissions. The empirical results show positive and significant coefficients of ln*AHSWeighted* (*α*_8_ > 0) in columns (1) and (2), indicating that higher effective tariffs on intermediate goods have significantly positive impacts on FEEE and DEEE.

Tables 4 and 5 list the GMM estimation results after adding relevant interaction terms. For FEEE, the estimated coefficients of the interaction terms are all significantly negative in columns (1) and (2), indicating that the improvement of economic complexity can decrease the marginal effects of export scale and export structure on FEEE significantly. In general, the increase in economic complexity could lead to a product space with lower carbon, thus reducing the overall FEEE. For DEEE, the interaction terms of eci*lnexport and eci**lnExportShare are negative but insignificant, indicating no indirect impacts of economic complexity on DEEE through the scale effect and the composition effect. Besides, the coefficients of eci*lnintensity are significantly positive for FEEE and DEEE, which means that the marginal positive impacts of technology can be further enhanced by economic complexity growth and the technological progress resulted from economic complexity improvement brings low carbon intensity. For FEEI, all the interaction terms have insignificant coefficients, thus rejecting Hypothesis b. This means that economic complexity cannot affect FEEI significantly through the proposed indirect mechanism. Meanwhile, economic complexity has significantly positive indirect impacts on DEEI through import scale, while no significant indirect impacts are found through composition effect and technology effect.

**Table 4:** Regression results with interaction terms for EEE

	(1)	(2)	(3)	(4)	(5)	(6)
	lnFEEE	lnFEEE	lnFEEE	lnDEEE	lnDEEE	lnDEEE
eci	0.718**	0.746**	0.283***	0.198	0.258	0.102**
	(2.13)	(2.50)	(3.33)	(1.11)	(1.54)	(2.26)
lnexport	0.680***	0.351***	0.557***	0.243***	0.159**	0.190***
	(6.46)	(3.55)	(5.78)	(3.04)	(2.40)	(2.91)
eci*lnexport	−0.0497*			−0.0152		
	(−1.68)			(−0.92)		
lnExportShare	0.536***	0.284***	0.446***	0.00629	−0.00635	−0.00320
	(4.93)	(3.39)	(4.95)	(0.20)	(−0.23)	(−0.14)
eci*lnExportShare		−0.165**			−0.0548	
		(−2.33)			(−1.37)	
lnintensity	0.254***	0.145***	0.0620	0.240***	0.180***	0.144**
	(3.82)	(2.72)	(0.88)	(4.02)	(3.06)	(2.28)
eci*lnintensity			0.195***			0.0763**
			(2.79)			(2.46)
Control variables	Yes	Yes	Yes	Yes	Yes	Yes
Sargan test	348.31***	284.02***	298.88***	156.70***	169.17***	153.07***
AR(1)	0.014	0.000	0.001	0.000	0.000	0.000
AR(2)	0.054	0.021	0.052	0.781	0.832	0.838
_cons	−8.021***	−4.132***	−6.682***	−1.978***	−1.221**	−1.541***
	(−6.18)	(−3.59)	(−5.75)	(−2.77)	(−2.27)	(−2.77)
*N*	580	580	580	580	580	580

1) ***, **, and * indicate that the coefficients are significant at the 1%, 5%, and 10% levels of significance, respectively. Numbers in brackets are *t* values

2) Sargan test checks the overidentification of instruments. Chi statistics are reported for the Sargan test, while the p-value is reported for AR(1) and AR(2)

**Table 5:** Regression results with interaction terms for EEI

	(1)	(2)	(3)	(4)	(5)	(6)
	lnFEEI	lnFEEI	lnFEEI	lnDEEI	lnDEEI	lnDEEI
eci	0.0569	0.695	0.0349	−0.452	0.540	0.0535
	(0.20)	(0.85)	(0.63)	(−1.63)	(1.24)	(1.40)
lnimports	0.421***	0.320***	0.393***	0.0687***	0.0762***	0.0587***
	(6.45)	(6.12)	(5.14)	(2.68)	(2.65)	(3.23)
eci*lnimport	−0.00898			0.0403*		
	(−0.35)			(1.69)		
lnImportShare	0.182	0.280	0.195	−0.0519	0.0246	−0.0456
	(1.04)	(1.53)	(1.10)	(−0.89)	(0.29)	(−1.12)
eci*lnImportShare		−0.171			−0.124	
		(−0.90)			(−1.22)	
lnintensity	0.106***	0.0943***	0.0730	0.0915**	0.0686**	0.0204
	(3.13)	(3.60)	(1.51)	(2.46)	(2.14)	(0.89)
eci*lnintensity			0.0558			0.0425
			(1.27)			(1.46)
Control variables	Yes	Yes	Yes	Yes	Yes	Yes
Sargan test	745.52***	260.87***	297.38***	326.24***	228.82***	225.05***
AR(1)	0.000	0.000	0.000	0.001	0.001	0.001
AR(2)	0.062	0.005	0.010	0.023	0.020	0.033
_cons	−3.967***	−3.589***	−3.814***	−0.420	−0.856	−0.409
	(−3.62)	(−4.01)	(−3.38)	(−1.19)	(−1.60)	(−1.54)
*N*	580	580	580	580	580	580

1) ***, **, and * indicate that the coefficients are significant at the 1%, 5%, and 10% levels of significance, respectively. Numbers in brackets are *t* values

2) Sargan test checks the overidentification of instruments. Chi statistics are reported for the Sargan test, while the p-value is reported for AR(1) and AR(2)

### Subsample regression results

Given the massive gap in political, economic, and social aspects between high-income countries and low- and middle-income countries, the estimation of Eqs. (1) and (2) may suffer from heterogeneity problems. Besides, there may exist a heterogeneous impact of economic complexity on EET in different country groups, which could even show a clear distinction in its impact direction. Therefore, it is necessary to regress economic complexity on embodied carbon emissions by country group.

Table 6 contains the subsample GMM estimation results for EEE and EEI. For high-income countries, the estimated coefficients of *ECI* are positive and significant in columns (1) and (2), but not significant in columns (3) and (4). This indicates that the FEEE and DEEE of industrialized countries would be further aggregated when high-income countries become more complex in production (higher ECI). Meanwhile, the promoting effect of economic complexity on FEEE is larger than that on DEEE. Therefore, high-income countries have a higher capability to embed more foreign carbon emissions in their exports, thus reaching a surplus in total EEE. This finding for high-income countries also supports the narrative that a more complex system could improve high-income countries’ environmental quality (Can and Gozgor 2017; Doğan et al. 2020).

**Table 6:** Subsample regression results

	(1)	(2)	(3)	(4)	(5)	(6)	(7)	(8)
	lnFEEE	lnDEEE	lnFEEI	lnDEEI	lnFEEE	lnDEEE	lnFEEI	lnDEEI
	High-income countries				Low- and middle-income countries			
eci	0.251**	0.0967**	0.0669	0.0224	0.0611	0.141**	0.0114	−0.0443**
	(2.35)	(2.11)	(1.55)	(1.24)	(0.71)	(2.28)	(0.16)	(−2.02)
lnexport	0.199**	0.0477			1.266***	0.249*		
	(2.17)	(1.01)			(5.83)	(1.93)		
lnExportShare	0.330**	−0.0696			0.372***	−0.0872*		
	(2.45)	(−1.27)			(3.42)	(−1.65)		
lnimports			0.143	−0.0327			0.490**	0.136**
			(1.46)	(−0.98)			(2.32)	(2.56)
lnImportShare			0.387**	−0.0289			0.466**	0.0258
			(2.27)	(−0.83)			(2.37)	(0.51)
lnintensity	0.0820	0.241***	0.174***	0.0501*	−0.259***	0.174**	0.0552	−0.0326
	(0.94)	(4.03)	(3.59)	(1.67)	(−3.10)	(2.17)	(0.80)	(−1.10)
Control variables	Yes	Yes	Yes	Yes	Yes	Yes	Yes	Yes
Sargan test	374.28***	165.33***	404.18***	155.08***	162.43***	93.52***	242.65***	111.33***
AR(1)	0.002	0.000	0.000	0.019	0.008	0.006	0.001	0.018
AR(2)	0.160	0.812	0.001	0.017	0.272	0.883	0.955	0.269
_cons	−6.812***	−3.439***	−8.693***	−0.655	2.723	−1.064	−8.740***	0.729
	(−3.11)	(−2.69)	(−4.66)	(−1.13)	(1.63)	(−0.86)	(−5.93)	(1.20)
*N*	360	360	360	360	220	220	220	220

1) ***, **, and * indicate that the coefficients are significant at the 1%, 5%, and 10% levels of significance, respectively. Numbers in brackets are *t* values

2) Sargan test checks the overidentification of instruments. Chi statistics are reported for the Sargan test, while the p-value is reported for AR(1) and AR(2)

In Table 6, columns (5)–(8) present the regression results for low- and middle-income countries. It can be seen that the estimated coefficients of *ECI* in columns (5) and (7) are positive but not significant. Therefore, present evidence of low- and middle-income countries fails to support a significant connection between economic complexity and FEEE and FEEI. However, the variable *ECI* has a significantly positive coefficient at the 5% level in columns (6) and a significantly negative coefficient at the 5% level in columns (8), respectively. These estimation results indicate that a one-unit increase in economic complexity could lead to a significant increase in DEEE by 14.1% and a decline in DEEI by 4.43% in the low- and middle-income countries group. The development of a more complex and knowledge-based economic system in low- and middle-income countries could alleviate the problem of CO_2_ transfer through their imports, but aggregate CO_2_ leakage problem through their exports, which is consistent with the findings of Wang et al. (2019).

In brief, the empirical results show asymmetric impacts of economic complexity on embodied CO_2_ emissions between high-income countries and low- and middle-income countries. The development of economic complexity in high-income countries could further deteriorate environmental quality by intensifying CO_2_ transfer through trade. However, low- and middle-income countries could reduce DEEI by improving their economic system towards a more complex structure. This finding is also consistent with the discussion of Yilanci and Pata (2020).

## Robustness checks

We provide a robustness analysis in this section to confirm the reliability of our primary empirical findings. The first method is to exclude outliers in our regression samples. When there are outliers in the data, the regression results are greatly affected by them because the estimation method treats them equally, and a more correct regression equation can not be given. The residual calculated based on this regression equation is naturally unreliable. As can be seen from Fig. 2, China and the USA exhibit extremely high carbon embodiment than other countries during the examined period. Panel A in Table 7 shows consistent estimation results as in Table 3—the estimated coefficients of eci are all significantly positive for lnFEEE, lnDEEE, and lnFEEI, but not for lnDEEI. The primary difference here is that the coefficients become higher after excluding the sample data of China and the USA. The second method is to estimate Eqs. (1) and (2) with another technique like dynamic fixed effect (DFE) technique with a pooled mean group (PMG). Panel B in Table 7 lists the long-run PMG estimators under DFE model, which yields consistent results for lnFEEE, lnFEEI, and lnFEEI, while the PMG estimator of lnDEEE becomes insignificant.

**Table 7:** Regression results after excluding outliers

	Panel A: GMM estimators excluding outliers				Panel B: DFE estimators with full sample			
	(1)	(2)	(3)	(4)	(1)	(2)	(3)	(4)
	lnFEEE	lnDEEE	lnFEEI	lnDEEI	lnFEEE	lnDEEE	lnFEEI	lnFEEI
eci	0.201**	0.177***	0.190***	0.0309	0.214**	0.125	0.192**	0.072
	(2.41)	(2.90)	(3.29)	(1.44)	(2.82)	(1.54)	(3.24)	(1.91)
lnexport	0.367***	0.0979			0.509**	0.116		
	(2.62)	(1.49)			(3.17)	(0.67)		
lnExportShare	0.459***	−0.0753			0.319**	0.229		
	(4.18)	(−1.47)			(2.80)	(1.88)		
lnimports			0.261**	0.0170			0.498***	0.194*
			(2.11)	(0.36)			(3.56)	(2.21)
lnImportShare			0.466**	−0.0417			0.765***	−0.029
			(2.53)	(−0.92)			(5.22)	(−0.31)
lnintensity	0.195**	0.543***	0.192***	0.0451**	0.467***	0.796***	0.210*	0.073
	(2.38)	(6.20)	(3.62)	(1.99)	(4.43)	(7.06)	(2.35)	(1.30)
Control variables	Yes	Yes	Yes	Yes	Yes	Yes	Yes	Yes
_cons	−7.987***	−8.137***	−12.07***	−0.975**	0.688	−6.001*	−6.519**	−0.637
	(−4.03)	(−5.15)	(−6.55)	(−2.12)	(0.21)	(−1.99)	(−3.16)	(−0.41)
*N*	560	560	560	560	580	580	580	580

1) ***, **, and * indicate that the coefficients are significant at the 1%, 5%, and 10% levels of significance, respectively. Numbers in brackets are *t* values

2) Panel A excluded the sample data of China and the USA under the estimation technique of GMM. Panel B employed the dynamic fixed effect model for estimation with all sample countries included

## Conclusions

This paper examines the impact of economic complexity on embodied carbon emissions based on panel data covering 58 countries from 2005 to 2015. There are limited studies that explore the relationship between economic complexity and embodied carbon emissions. Our paper contributes to the existing literature by adding this variable to our econometric model. We utilized the latest GMM technique to estimate the determinants of embodied carbon emissions and found that the impact of economic complexity on trade-embodied carbon emissions is heterogeneous. Specifically, economic complexity has significant positive impacts on FEEE, DEEE, and FEEI in the pooled sample. A one-unit rise in economic complexity can increase FEEE, DEEE, and FEEI by 18.8%, 16.5%, and 16.4%. We found no evidence of significant impacts of economic complexity on DEEI in our pooled sample. Besides, the scale effect, structural effect, and technological effect are also significant drivers of embodied carbon emissions. As for the impact mechanism, the improvement of economic complexity can decrease the marginal effects of export scale and export structure on FEEE (but not DEEE) significantly, while the marginal positive impacts of technology on FEEE and DEEE can be further enhanced by economic complexity growth and the technological progress resulted from economic complexity improvement brings low carbon intensity. Besides, there is no strong evidence to prove the significant indirect impacts of economic complexity on FEEI, while economic complexity has significantly positive indirect impacts on DEEI only through import scale. In the subsample regressions, we found asymmetric impacts of economic complexity between high-income countries and low- and middle-income countries. The FEEE and FEEI of high-income countries are all positively affected by economic complexity, which are significant in statistics. There is no sufficient evidence to support a significant relationship between economic complexity and EEI in high-income countries. However, developing a more complex production system in low- and middle-income countries would decrease their DEEI significantly by 4.43% and increase their DEEE significant by 14.1%. For low- and middle-income countries, we found no evidence of the significant impact of economic complexity on foreign carbon embodiment.

This paper’s empirical findings also provide some policy implications as follows: first, when defining the scope of responsibilities of carbon emissions, a more integrated platform needs to be provided in the national society to take global CO_2_ transfer through trade into consideration as the amount of embodied carbon emissions in exports and imports have been increasing in some countries in recent years. Second, the improvement of the economic system may bring heterogeneous outcomes to the country’s trade-embodied carbon emissions, depending on its overall economic, political and social conditions. For high-income countries, their policymakers should support the development of more environmental-friendly technologies and provide necessary aid to other developing countries to improve their environmental quality. For low- and middle-income countries, to alleviate CO_2_ leakage, it is essential to improve the economic complexity and produce more knowledge-based products. Finally, policymakers could also reduce trade-embodied carbon emission by reducing carbon emission intensity, designing favorable tariff policies, and transforming the structure of traded goods.

This study contributed new evidence in the relationship between economic complexity and trade-embodied carbon emissions. Future studies can make innovative research by incorporating other interesting variables like financial stability, institutional quality, labor cost, and environmental policy in the econometric models.

## Data Availability

The data that support the findings of this study is available for sharing from corresponding author upon reasonable request.

## Appendix

**Table 8:** List of sample countries/regions

High-income countries		Low and middle-income countries
AUS: Australia	NZL: New Zealand	ARG: Argentina
AUT: Austria	NOR: Norway	BRA: Brazil
BEL: Belgium	POL: Poland	BGR: Bulgaria
CAN: Canada	PRT: Portugal	KHM: Cambodia
CHL: Chile	SAU Saudi Arabia	CHN: China
HRV: Croatia	SVK: Slovakia	COL: Colombia
HKG: Hong Kong SAR	SVN: Slovenia	CRI: Costa Rica
CZE: Czech Republic	KOR: South Korea	IND: India
DNK: Denmark	ESP: Spain	IDN: Indonesia
EST: Estonia	SWE: Sweden	KAZ: Kazakhstan
FIN: Finland	SGP: Singapore	MYS: Malaysia
FRA: France	CHE: Switzerland	MEX: Mexico
DEU: Germany	GBR: United Kingdom	MAR: Morocco
GRC: Greece	USA: United States	PER: Peru
HUN: Hungary		PHL: Philippines
IRL: Ireland		ROU: Romania
ISR: Israel		RUS: Russia
ITA: Italy		TUR: Turkey
JPN: Japan		ZAF: South Africa
LVA: Latvia		THA: Thailand
LTU: Lithuania		TUN: Tunisia
NLD: Netherlands		VNM: Vietnam

**Table 9:** Profiles of the explained variables (for the manufacturing industry)

Variable	Explanation
lnFEEE	Logarithm of foreign CO_2_ emissions embodied in gross exports
lnDEEE	Logarithm of domestic CO_2_ emissions embodied in gross exports
lnDEEI	Logarithm of domestic CO_2_ emissions embodied in gross imports
lnFEEI	Logarithm of foreign CO_2_ emissions embodied in gross imports

## Code availability

Statistical codes used in this research are available for sharing from corresponding author upon reasonable request.
